# Priapism in a 28-year-old Male with Bipolar Disorder

**DOI:** 10.7759/cureus.7721

**Published:** 2020-04-18

**Authors:** Eduardo D Espiridion, Zachary Danssaert, Robert Libera

**Affiliations:** 1 Psychiatry, Reading Hospital - Tower Health, West Reading, USA; 2 Psychiatry, Drexel University College of Medicine, Philadelphia, USA; 3 Psychiatry, West Virginia School of Osteopathic Medicine, Lewisburg, USA; 4 Psychiatry, West Virginia University School of Medicine, Martinsburg, USA; 5 Psychiatry, Philadelphia College of Osteopathic Medicine, Philadelphia, USA; 6 Medicine, Philadelphia College of Osteopathic Medicine, Philadelphia, USA; 7 Internal Medical, Philadelphia College of Osteopathic Medicine, Philadelphia, USA

**Keywords:** priapism, topiramate

## Abstract

Priapism is a serious but rare side effect of psychiatric medications. We report the case of a 28-year-old male who has been mentally stable for a year on a combination of sertraline, olanzapine, and quetiapine. Two weeks after he was started on topiramate, the patient experienced priapism that led to a medical admission and surgical intervention. This case report suggests that topiramate might induce ischemic priapism, either directly or through interactions with other medications. Further studies are warranted to establish such a link.

## Introduction

Priapism is defined as a prolonged painful penile or clitoral erection that may last for more than four hours and is not associated with sexual stimulation. Penile priapism is a rare condition, with an incidence of 0.73 cases per 100,000 men per year, with many of the cases occurring in young men between the ages of 20 to 50 years old [1,2]. There are two major categories of priapism: ischemic and non-ischemic. Non-ischemic priapism is a self-resolving condition that only occurs in the setting of direct trauma to the penis, and the erection is not painful or rigid [3]. In contrast, ischemic priapism presents with an acutely painful and rigid erection requiring emergent treatment to prevent fibrosis and long-term erectile dysfunction [4]. Recurrent, or stuttering, priapism is a sub-type of ischemic priapism whereby patients present with erections of a short duration that increase in frequency, leading to an event of ischemic priapism. Most cases of priapism are idiopathic, but there have been drugs that have demonstrated to cause the condition.

## Case presentation

A 28-year-old male patient was admitted to the medical floor of a community hospital due to a painful erection of three days’ duration. He had not experienced this before. The erection was unprovoked and had occurred spontaneously. There was no sexual stimulation involved. He complained of pain in the legs and testicles and he was unable to urinate. The pain was described to the nursing staff as "severe", but no pain scales were used to measure the intensity. There was penile swelling and tenderness on examination. He denied any current illicit drug use, including cocaine and methamphetamine. The patient denied any perineal or penile trauma. He does not have a history of sickle cell anemia, thalassemia, or multiple myeloma. He was not using any medications for erectile dysfunction. While in the emergency room, the patient was afebrile and hemodynamically stable. The base of his penis was cleansed with iodine and anesthetized. The patient underwent aspiration and irrigation followed by intracorporeal phenylephrine injection to achieve detumescence. Approximately 100 mL of blood was removed. The patient tolerated well the procedure, and he noted that the penile pain improved immediately, but the penis remained erect. A Urologist was subsequently consulted by the attending physician. There was no documentation of necrosis, and his penis was no longer erect three hours later. There was no penile edema or ecchymosis observed. There was no further intervention recommended afterward. He reported to his outpatient therapist a week later that he has not experienced erectile dysfunction or recurrence of the priapism. He was scheduled to have a follow-up visit with the urologist who treated him.

During the psychiatric consultation, the patient was calm and cooperative. He denied any depressive, manic, or psychotic symptoms. He was anxious because of his pain symptoms. There were no suicidal or homicidal thoughts. His psychiatric home medications, which included olanzapine, quetiapine, and sertraline, were held. The patient reported a prior psychiatric history and prior psychiatric hospitalization. The patient admitted to a history of mood swings, irritability, rage reactions, sleeping disturbances, racing thoughts, poor impulse control, and psychomotor agitation. He had been taking his home medications for five months. He was compliant with his medication regimen and treatment follow-ups. He was diagnosed with bipolar disorder after a psychiatric hospitalization the year before. He had not experienced any painful erections since his psychiatric medications were started. Because of a recent argument with his girlfriend, the patient experienced worsening irritability. His physician assistant started him on topiramate 50 mg twice a day. He took a week’s dose of topiramate when he noticed the painful erections.

## Discussion

The patient presented with a medical history of bipolar disorder medically managed for five months with olanzapine, quetiapine, and sertraline. For the past seven days, topiramate 50 mg twice a day was added to his regimen, which likely precipitated his persistent painful erection. Priapism is a pathologic penile emergency characterized by a prolonged state of erection in the absence of sexual arousal [5]. It is a compartment syndrome that requires immediate intervention to avoid necrosis of the cavernous muscle, subsequent fibrosis, and erectile dysfunction [6]. The American Urological Association recommends a stepwise treatment protocol for ischemic priapism starting with injection of a sympathomimetic agent, with phenylephrine being the agent of choice. Repeated injections are recommended before surgical interventions [3].

Psychotropic medication-inducing priapism exert alpha adrenergic blockade, which enhances parasympathetic activity to sustained erections. The antidepressants such as phenelzine and trazodone, as well as antipsychotics such as chlorpromazine, thioridazine, phenothiazines, fluphenazine, mesoridazine, haloperidol, and molindone have been associated with priapism [7]. Other vasoactive medications such as hydralazine, prazosin, alcohol, and cocaine may lead to ischemic priapism [5].

Topiramate's effect on sexual function has mostly been associated with erectile dysfunction and libido disorder in male patients [8]. The etiology of the topiramate-induced erectile dysfunction is not well understood, but reproductive hormones are not affected by the drug, which points to a vasogenic action as the possible underlying cause [9]. A known side effect of topiramate is metabolic acidosis, which can subsequently cause the release of nitric acid to dilate the arterioles [10-11]. Indeed, a vasogenic mechanism would help explain topiramate's ability to cause erectile dysfunction and ischemic priapism. However, the occurrence of priapism associated with topiramate is not well documented.

There has been a reported case of a reversible and stuttering priapism in association with topiramate [12]. The patient developed episodic priapism soon after taking topiramate, which went away after discontinuing the medication. In another case report, a patient experienced ischemic priapism while taking olanzapine and topiramate together, but the patient was experiencing episodic erections while on olanzapine alone, which was not altered by the addition of the topiramate. Although the patient had reversible priapism while on both medications, olanzapine was thought to be the culprit [13].

Another important point to highlight in the treatment of ischemic priapism is the urgency and timeliness of the medical interventions to avoid any significant complications. Patients with this dilemma may unnecessarily delay treatment and care due to the embarrassment of the medical complaint. Patients with genital lesions experience greater feelings of shame and lower self-esteem compared with other medical issues [14]. This may be the reason why this patient waited three days before he went to the emergency department. He delayed the treatment for this malady until the time the pain became unbearable.

This case is unique because ischemic priapism appears to be in direct relation to topiramate. We cannot exclude the possibility that the priapism was associated with his other psychiatric medications or if there were synergistic activities. The patient experienced ischemic priapism shortly after topiramate was added to his medication regimen. Therefore, topiramate may have caused the priapism directly or it interacted with his other psychiatric medications.

## Conclusions

There is a lack of documentation of topiramate's association with priapism. We have presented the case of a patient taking multiple psychiatric medications, which individually may have precipitated the priapism. However, the addition of topiramate was most likely the cause either directly or through interactions with his other medications. Further documentation is needed to explore the relationship between topiramate and priapism.
